# Assessing advances in regional anesthesia by their portrayals in meta-analyses: an alternative view on recent progress

**DOI:** 10.1186/s12871-017-0406-3

**Published:** 2017-08-29

**Authors:** Kamen V. Vlassakov, Igor Kissin

**Affiliations:** 1 Department of Anesthesiology, Perioperative and Pain Medicine, Brigham and Women’s Hospital, Harvard Medical School, Boston, MA USA; 2 Department of Anesthesiology, Brigham and Women’s Hospital, 75 Francis St, Boston, MA 20115 USA

**Keywords:** Epidural anesthesia, Spinal anesthesia, Nerve blocks, Minimal clinically important difference, Real-world evidence

## Abstract

**Background:**

The aim of this study was to delineate research reflecting advances in regional/local anesthesia where recent clinical progress was clearly defined by meta-analysis.

**Methods:**

We conducted a search to identify all articles with meta-analyses of randomized clinical trials related to the field of regional/local anesthesia. From 279 titles, after multiple exclusions, 16 meta-analyses on important clinical practice developments with high potential for a positive conclusion on the effectiveness of the treatment were left for the assessment. The assessment was performed in two steps. The first step was related to verification of proof-of-concept: the effect is statistically reliable (*p*-value, effect size, heterogeneity across different RCTs) and the risk of bias not too high. The second step was devoted to attempts to form an opinion on the real clinical benefits of a new development.

**Results:**

The assessment revealed that seven recent developments passed the proof-of-concept step. At the same time, positive conclusion on real clinical benefits was reached only by one of these seven developments: ultrasound guidance for peripheral nerve blocks (at least with some of the blocks). Meaningful clinical improvements with other developments remains uncertain. The assessment of the relationships between analyzed advancements over the past 30 years and earlier similar developments indicated that their evolution was usually incremental. The most original advancement was found to be the introduction of the transversus abdominis plane block.

**Conclusion:**

The assessment of recent advances in regional/local anesthesia, based on the evaluation of related meta-analyses, revealed only incremental progress with mostly marginal benefits. The progress was the most notable with ultrasound guidance for some of peripheral nerve blocks.

## Background

In our previous study [1] we evaluated the evolution of different anesthetics and techniques of their administration using a number of scientometric indices and concluded that, for the past 30 years, there have been no significant advances that have produced changes in these indices indicating real progress. It is of interest how the conclusions on clinical progress look when the outcomes of research efforts are assessed using the methods of evidence-based medicine reflected by meta-analysis. Scientometric indices showed that in the last three decades the most significant academic efforts in anesthesia were concentrated on investigations related to the use of local anesthetics and the least significant – on inhalational anesthetics [1]. In this study, we summarize the outcomes of efforts related to regional/local anesthesia as seen through meta-analysis. The aim of this study was to delineate research advances in regional/local anesthesia where recent clinical progress was clearly defined by meta-analysis.

## Methods

The initial search of meta-analyses was performed with the goal of identifying articles describing the analysis of randomized clinical trials (RCT) related to the field of regional/local anesthesia. The search was conducted using both electronic and manual methods. In the electronic search, we used the PubMed database, i.e., the National Library of Medicine’s PubMed website (http://www.ncbi.nlm.nih.gov/pubmed) including the Cochrane Database of Systematic Reviews (CDSR). The following “MeSH terms” were entered into the search box: “Anesthesia, Epidural” OR “Anesthesia, Spinal” OR “Anesthesia, Local” OR “Nerve Block”, as well as a non-MeSH term “regional anesthesia”. In addition, “Meta-Analysis” was used as the PubMed filter for the type of article. The filter for languages (English) was activated. In manual searches, we used the reference lists of the relevant articles with meta-analyses found in the above mentioned electronic searches.

**Fig. 1:**
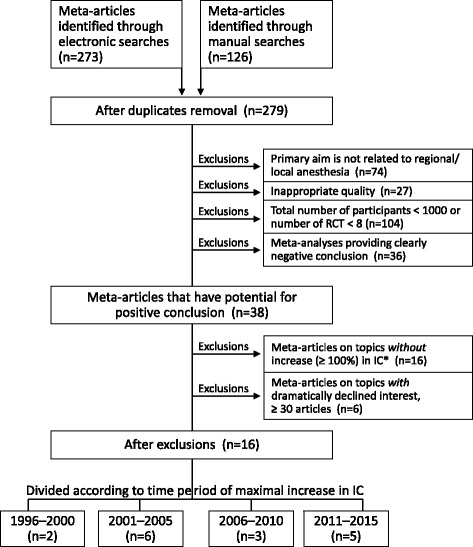
Flowchart of screened, excluded and included articles with meta-analysis

All abstracts generated by the aforementioned searches were reviewed to determine that the primary aim was related to regional/local anesthesia; all other meta-analyses were excluded (Fig. 1, exclusion 1). Further exclusions were based on inspections of full-text articles. The aim of the exclusions was to select meta-articles with real potential for a positive conclusion on the effectiveness of the treatment. In the assessment of treatment effectiveness we relied on the conclusions presented by the authors of meta-analysis. First, if they provided a clearly negative conclusion on the treatment effectiveness, it was the basis for the exclusion of a meta-analysis from the list of articles that has the potential for a positive conclusion (Fig. 1, exclusion 4). Second, we presented the results of meta-analyses as they were determined by the authors, we only graded the magnitude of the obtained results for the sake of valid comparisons. The following three additional exclusion criteria were used: Inappropriate research quality, such as non-original quantitative research, absence of the statistical procedure for combining data from multiple studies, inclusion of non-randomized trials, or the comparison of single doses of two (or several) drugs (Fig. 1, exclusion 2); The total number of participants included in a meta-analysis was <1000, or the number of RCTs for a selected outcome was <8 (Fig. 1, exclusion 3). The use of the minimum of 1000 participants in total was based on the recommendation by Humaidan and Polyzos [2]. The recent review of meta-analyses of anesthesiologic interventions indicated that the median number of participants was very close to 1000–964, and the median number of included trials was 8 [3].

Because the aim of the study was to analyze important advances in regional anesthesia, we also eliminated meta-analyses associated with topics of no primary importance. To achieve this, the scientometric index of change (IC) was used to grade the increase in academic popularity of a related advancement. The IC represents the degree of growth in publication on a topic from one period to the next; it has been shown to be a good indicator of academic interest in a topic [1]. The number of articles on a topic during a 5-year period was compared (percentage change) with the previous 5-year period. For this aim, the topic of the meta-article was entered into the PubMed search box (for example,- “ultrasound-guided nerve block”) and the number of articles published over five 5-year periods (1991–1995, 1996–2000, 2001–2005, 2006–2010, and 2011–2015) was counted. Filter for languages (English) was used, and all types of articles were considered. Exclusions based on the character of change in academic interest in the topic were of two types (Figure 1). The first exclusion, based on academic popularity, were related to the degree of IC increase: meta-articles on topics without important IC increase (≥100%) in any of the 5-year periods were excluded as failing to generate interest adequate for a significant advancement. The second exclusion was based on a dramatic decline in the interest in the topic during the last 5-year period (2011–2015), i.e., fewer than 30 articles published during that period (any type of articles).

Selected meta-analyses were divided into distinct topics. In each selected topic only one outcome was presented in the table with mostly statistical values determined by the authors of meta-analysis, such as number of participants with outcome, *P*-value, effect size, heterogeneity, and risk of bias*.* When there were several meta-analyses on a topic, only the meta-article with the most statistically reliable values was presented in the table with specific statistics, other selected meta-analyses on the same topic were presented separately.

The assessment of new developments in regional anesthesia was performed in steps. The first step was related to verification of proof-of-concept: the effect is statistically significant and the risk of bias not too high. This assessment was based on the results obtained by the authors of meta-analyses regarding the following criteria: the degree of statistical significance (*p* value), the magnitude of difference between compared groups (effect size), and the degree of heterogeneity across different RCTs summarized in the meta-analysis. The degree of statistical significance was used to avoid skepticism that a certain precise *P* value is not a best practice for hypothesis testing [4, 5]. We used the effect size as determined by the authors of respective meta-analyses, but for the sake of valid comparison we graded its degree: small, medium, or large [6]. The heterogeneity across summarized RCTs was also used as determined by the authors of respective meta-analyses; most commonly, it was I^2^ statistics. We only graded its degree as low (I^2^ < 50%), moderate (I^2^ = 50% – 75%), or high (I^2^ > 75%) [7]. Another category of the evaluation was associated with the risk of bias. It was usually based on the Cochrane Collaboration principles [8] determined by the authors of the meta-analyses, and graded as high, moderate, or low.

An incomparably more difficult step in the assessment of a new development is a conclusion on its real clinical benefits. Challenges in determining clinical significance of any improvement have led to the development of a new type of indicator – minimal clinically important difference (MCID) [9–11]. This indicator requires the development of specific methodology for every topic. A MCID index has been developed for the intensity scores of acute pain [10, 11], but not for the other outcomes in the assessed meta-analyses. In an attempt to look beyond the proof-of-concept evaluation --into the assessment of real clinical benefits, we tried to use the concept of MCID when the index was available (for scores of acute pain). The other approach was based on the assessment of the conclusions of the authors of positive meta-analyses on the real clinical benefits, if important problems related to them were clearly stated. As a result, the considerations related to this most important step of the development assessment were summarized in a specific table.

In an additional step, in order to estimate the degree of novelty of each new development, its relationships to earlier similar developments were traced and assessed.

## Results

Our searches yielded 279 titles (Fig. 1), 74 of which were excluded because their primary aims were not directly related to regional/local anesthesia. Twenty-seven articles were excluded due to the inappropriate quality of their meta-analyses. The largest number of exclusions – 104 was made due to the insufficient total number of participants or insufficient number of RCTs. In addition, 36 meta-articles provided clearly negative conclusions, excluding the possibility for positive assessment of an advancement in regional/local anesthesia. At the end of the process, 38 articles remained in the flowchart.

Because the aim of our study was to analyze important advances in regional anesthesia, a number of additional exclusions were based on the assessment of scientometric index of change (IC) that reflects the academic interest in a topic. With this aim we determined the IC for the topics of meta-analyses under review (Table 1). Sixteen meta-analyses with IC < 100% were excluded (Fig. 1). In addition, six meta-articles were excluded because of a decline in the academic interest in a topic during the last 5-year period (2011–2015) to fewer than 30 articles. After all exclusions, 16 meta-analyses related to the eight topics were left for the assessment (Table 2). Divided according to the time period of maximal increase in the IC (Table 1), they include: for 1996–2000 – two meta-articles, for 2001–2005 – six meta-articles, for 2006–2010 – three meta-articles, and for 2011–2015 – five meta-articles (Fig.1).

**Table 1:** Topics of selected meta-analyses classified according to time of rise in the academic interest (IC)^a^ to related area

Topic	Total number of area articles	Degree of rise in IC			
	1996–2015	1996–2000	2001–2005	2006–2010	2011–2015
Thoracic paravertebral block in breast surgery	49	_	_	_	++++
Perineural dexamethasone as adjunct for peripheral nerve block	83	_	_	_	++++
Transversus abdominis plane block	211	_	_	+++	+++
Ultrasound guidance for peripheral nerve blocks	789	_	+++	++++	+
Femoral nerve block in knee surgery	251	_	+++	_	+
Local anesthesia for prostate biopsy	125	_	++++	_	_
Epidural anesthesia combined with general anesthesia for cardiac surgery, impact on mortality and morbidity	161	++	+++	_	_
Transient neurologic symptoms in spinal anesthesia	96	++++	_	_	_
Preemptive effect of epidural analgesics	165	++++	_	_	+
Topic	Total number of area articles	Degree of rise in IC			
	1996–2015	1996–2000	2001–2005	2006–2010	2011–2015
Spinal anesthesia and intraoperative blood loss	130	++	_	_	_
Neostigmine in neuraxial anesthesia	96	++	_	_	_
Local anesthesia for hysteroscopy	62	++	_	_	_

^a^IC, is the percentage change in the number of articles on a topic during a 5-year period compared with the previous similar period

+ − IC = 0–100%, ++ ˗ IC > 100%, +++ ˗ IC > 200%, ++++ ˗ IC > 400%

**Table 2:** Topics of meta-analyses after their final selection

#	Topic	Time period of maximal rise in IC	Number of selected meta-analyses	Authors
1	Thoracic paravertebral block in breast surgery	2011–2015	1	Terkawi et al., 2015 [12]
2	Perineural dexamethasone as adjunct for peripheral nerve block	2011–2015	3	**Albrecht et al., 2015** [13]**;** Huynh et al. 2015 [14]; Knezevic et al., 2015 [15]
3	Transversus abdominis plane block	2011–2015	1	Baeriswyl et al., 2015 [16]
4	Ultrasound guidance for peripheral nerve blocks	2006–2010	3	**Lewis et al., 2015**; [17] Munirama and McLeod 2015; [18] Gelfand et al., 2011 [19]
5	Femoral nerve block in knee surgery	2001–2005	3	**Chan et al., 2014** [20]; Xu et al. 2014 [21]; Paul et al., 2010 [22]
6	Effect of perioperative epidural analgesia combined with general anesthesia on mortality in cardiac surgery	2001–2005	3	Zhang et al., 2015; [23] **Svircevic et al., 2013**; [24] Bignami et al., 2010 [25]
7	Preemptive effect of epidural analgesics	1996–2000	1	Ong et al., 2005 [26]
8	Spinal anesthesia and intraoperative blood loss	1996–2000	1	Richman et al., 2006 [27]
Total	8		16	-

In bold letters are the meta-articles with the outcomes selected for the assessment when there were several meta-analyses on a topic (only the most reliable outcome of all the meta-articles on the topic was selected)

Sixteen meta-analyses, presented in Table 2 [12–27], reflect four topics with one meta-analysis and four other topics, each with three meta-analyses. In each of the topics only one representative study (Table 3) has its statistical values included in Table 4. It represents the first step in the assessment of the selected outcomes: whether an outcome passed the proof-of-concept validation, i.e., if the effect is statistically significant and the risk of bias not too high. Table 4 presents the related data. It indicates that with one of the outcomes the statistical power was too low to reach statistical significance – “the effect of perioperative epidural analgesia combined with general anesthesia on mortality in cardiac surgery.” The *p* value was insufficient despite a very high number of participants with the results – 2877. All other outcomes had high levels of statistical significance. Table 4 also demonstrates that the high heterogeneity (across different RCTs summarized in the meta-analyses) in general represents a problem with the certainty of conclusions based on these analyses. High levels of heterogeneity were observed with most of the outcomes; and only one outcome – “ultrasound guidance for upper and lower limb nerve blocks” – had low heterogeneity. As far as the risk of bias is concerned, only two of the assessed outcomes had problems with it. One of these outcomes –“perineural dexamethasone as an analgesic adjunt for peripheral nerve block” – had a high risk of bias. With another outcome – “the spinal anesthesia and intraoperative blood loss” (Richman et al. study [27]) – the risk of bias was not properly assessed. In general, the proof-of-concept mainly represents statistical confirmation that the difference between an effect and a control is real. Conclusions regarding this first step in the assessment of a development are presented in the first column of Table 6. Only one of the eight assessed outcomes – “the effect of perioperative epidural analgesia combined with general anesthesia on mortality in cardiac surgery” – did not pass this step in the assessment.

**Table 3:** List of selected outcomes

**#**	Authors	Selected outcome
1	Terkawi et al., 2015 [12]	Thoracic paravertebral block for breast surgery reduced postoperative pain score at rest at 24 h compared to no intervention.
2	Albrecht et al., 2015 [13]	Perineural dexamethasone increased duration of postoperative analgesia, defined as time to first analgesic request, when used with long-acting local anesthetics.
3	Baeriswyl et al., 2015 [16]	Ultrasound-guided transversus abdominis plane block reduced cumulative morphine consumption at 6 h postoperatively after abdominal surgery with general or spinal anesthesia.
4	Lewis et al., 2015 [17]	Ultrasound guidance increased the upper and lower limb nerve block success rate defined by the lack of need for analgesic or anesthetic rescue.
5	Chan et al., 2014 [20]	Femoral nerve block improved analgesia, defined as decrease in pain score at rest at 24 h postoperatively, as compared with systemic opioids (patient-controlled analgesia).
6	Svircevic et al., 2013 [24]	Thoracic epidural anesthesia given in combination with general anesthesia reduced the risk of mortality in patients undergoing cardiac surgery as compared with general anesthesia alone.
7	Ong et al., 2005 [26]	Preincisional administration of epidural analgesics decreased postoperative (24–48 h) pain scores to a greater degree than similar postincisional analgesic interventions.
8	Richman et al., 2006 [27]	Spinal anesthesia was associated with lower estimated intraoperative blood loss when compared to general anesthesia.

**Table 4:** Statistics and risk of bias for selected outcomes

Outcome#	Authors	Trials	Participants with outcome	Statistical Analysis			Risk of bias^c^
				*P*-value	Effect size^a^	Heterogeneity^b^	
1	Terkawi et al., 2015 [12]	21	1714	*p* < 0.00001	Medium	High	Moderate
2	Albrecht et al., 2015 [13]	18	566	*p* < 0.00001	Large	High	High
3	Baeriswyl et al., 2015 [16]	18	886	*p* < 0.00001	Large	High	Moderate
4	Lewis et al., 2015 [17]	18	1807	*p* < 0.00001	Large	Low	Moderate
5	Chan et al., 2014 [20]	9	416	*p* = 0.00007	Large	Moderate	Moderate
6	Svircevic et al., 2013 [24]	31	2877	*p* = 0.72	–	–	–
7	Ong et al., 2005 [26]	13	653	*p* = 0.002	Medium	High	Moderate
8	Richman et al., 2006 [27]	14	NA	*p* < 0.00001	Medium	NA	NA

^a^Degree was graded as small, medium, or large according to Sullivan and Feinn [6]

^b^Degree was graded as low, moderate, or high [7]

^c^The authors’ of respective meta-analysis conclusion, based on the Cochrane Collaboration principles [8], graded as high, moderate, or low

A much more difficult step in the assessment of a new development is a conclusion on the clinical importance of an achieved improvement. It is task that requires the development of specific MCID indices for every selected outcome (see Methods). Table 5 presents comments related to clinical importance; they mostly represent opinions, not proofs. Our summary on the real clinical benefits (second column in Table 6) is based on these comments. The column demonstrates two definite negative assessments, five assessments with question marks, and only one positive assessment.

**Table 5:** Comments related to clinical importance

#	Topic	Problems
1	Thoracic paravertebral block to provide analgesia in breast surgery	With the rating scale from 0 to 10.0, the maximal decrease in pain intensity of 0.89 (1.29; 0.49)--determined in the related meta-analysis-- was less than the minimal clinically important improvement with pain of moderate intensity: ≥ 1.9 [10]. The comment of authors of the meta-analysis – “Thoracic paravertebral block has a limited beneficial effect on quality of recovery”-- confirms that the proof of meaningful clinical improvement due to this intervention was not convincing.
2	Perineural dexamethasone as an analgesic adjunct for peripheral nerve block	The perineural administration of dexamethasone seems to provide only modest and inconsistent addition to its systemic effect on the duration of postoperative analgesia [28, 29]. Thus the observed effect is mostly due to the systemic analgesic effect of dexamethasone described long ago. The authors of the meta-analysis made a similar comment in this regard.
3	Transversus abdominis plane block to provide analgesia in abdominal surgery	The block-induced reduction in postoperative morphine consumption was so modest that the authors of the meta-analysis made the following comments on the clinical importance of the outcome: “Marginal analgesic efficacy”... “Clinical impact is questionable”. A problem could be seen in the very high heterogeneity of the related analysis (I^2^ = 94%), probably due to inclusion in the analysis of very different types of surgical procedures and many other outcome variables. In addition, there was no appropriate comparison to other well-established analgesic regimens [32].
4	Ultrasound guidance for peripheral nerve blocks	The absence of major problems with the meaningful clinical improvement resulting from this technique is reassuring. In addition, the related meta-analysis has an exceptionally low degree of heterogeneity (I^2^ = 16%).
5	Femoral nerve block to provide analgesia in knee surgery	With the rating scale from 0 to 10.0, the maximal decrease in postoperative pain intensity of 0.72 (0.93; 0.51) determined in the related meta-analysis was less than the minimal clinically important improvement with pain of moderate intensity ≥ 1.9 [10]. In addition, the improvement was observed only when compared with IV PCA, not with the other common methods of analgesia (epidural or local infiltration).
6	Effect of perioperative epidural analgesia combined with general analgesia on mortality in cardiac surgery	The statistical power was too low to reach even statistical significance for beneficial effect estimate.
7	Preemptive effect of epidural analgesics	The authors of the related meta-analysis expressed the effect on pain only as a value that has no units, therefore the real pain score change is difficult to assess. However, the effect size of the observed difference was graded as medium, not large (Table 4). Continuing controversy regarding degree of clinical effectiveness of preemptive anesthesia makes it difficult to come to definite conclusion on the clinical importance related to this intervention.
8	Spinal anesthesia is associated with lower intraoperative blood loss	The assessment of the clinical importance of this meta-analysis result is weakened by the absence of data on the risk of bias, heterogeneity, and the inclusion of very old RCTs starting in 1972. The meaningful clinical importance was not quite obvious to the authors of this meta-analysis, who commented: “Unclear that this finding is clinically meaningful, e.g. with result in a reduction of blood transfusion.”

**Table 6:** Two-step evaluation of new developments in regional anesthesia

	Topic	Steps in assessment	
		Proof-of-concept: effect is statistically significant^a^	Opinion on real clinical benefits^b^
1	Thoracic paravertebral block to provide analgesia in breast surgery	Yes	?^c^
2	Perineural dexamethasone as an analgesic adjunct for peripheral nerve block	Yes	No
3	Transversus abdominis plane block to provide analgesia in abdominal surgery	Yes	?^c^
4	Ultrasound guidance for peripheral nerve blocks	Yes	Yes^d^
5	Femoral nerve block to provide analgesia in knee surgery	Yes	?^c^
6	Effect of perioperative epidural analgesia on mortality in cardiac surgery	No	No
7	Preemptive effect of epidural analgesics	Yes	?^c^
8	Spinal anesthesia is associated with lower intraoperative blood loss	Yes	?^c^

^a^Based on analysis presented in Table 4

^b^Based on comments presented in Table 5

^c^Questionable due to the problems listed in Table 5

^d^At least with upper and lower limb blocks

Here we present four topics with more than one meta-analysis (Table 2). One of them – “the effect of perioperative epidural analgesia combined with general anesthesia on mortality in cardiac surgery” – did not pass the proof-of-concept test: *P*-value was >0.05 in any of the 3 included meta-analyses [23–25]. Another topic – “perineural dexamethasone as an analgesic adjunct for peripheral nerve block” – has a definite possibility that this effect is mostly due to the systemic analgesic action of dexamethasone [28, 29] described long ago. All three meta-analyses confirmed [13–15] that such possibility is present. Two multiple meta-analysis topics (in our evaluation without clearly negative outcomes, Table 6) are – “femoral nerve block to provide analgesia in knee surgery” and “ultrasound guidance for peripheral nerve blocks.” Two [20, 21] of the three meta-analyses on the femoral nerve block have the structured risk of bias assessment. Although only one [20] of them was presented in Tables 4, both are of almost equal quality. As far as the topic of ultrasound guidance is concerned, of the three meta-analyses only one [17] had a very low degree of heterogeneity and was selected for the inclusion in the Tables 4.

Table 7 represents the assessments of the relationships between analyzed advancements over the past 30 years and earlier similar developments. With almost all topics, progress in the development of new advances in regional anesthesia was incremental. The only really novel development was the introduction of the transversus abdominis plane block (O’Donnell et al. [30]).

**Table 7:** Relationship of analyzed topics with earlier developments

#	Topic	Time period of initial publications	Relationships with earlier developments
1	Thoracic paravertebral block to provide analgesia in breast surgery	1995–1996 [46, 47]	Paravertebral blocks were first performed in 1905 [48].Their use for post-thoracotomy and traumatic chest pain, and also for permanent neurolytic block was described as early as 1962. Resurgence of interest in paravertebral blocks was based initially on their favorable reappraisal in 1979 by Eason and Wyatt [49], but subsequently on the relative hemodynamic stability compared to the dominant neuraxial block techniques.
2	Perineural dexamethasone as an analgesic adjunct for peripheral nerve block	2004–2010 [50, 51]	It was recently demonstrated that systemic dexamethasone may be equivalent to perineural dexamethasone in prolonging the analgesic duration of local anesthetic nerve blockade, and a perineural mechanism of its action provides only a modest and inconsistent supplementation to this prolongation [28, 29]. The effect of systemic glucocorticoids on postoperative analgesia has long been known. These effects cannot be explained only by their anti-inflammatory actions; their analgesic efficacy may be independent of their anti-inflammatory actions [52].
3	Transversus abdominis plane block to provide analgesia in abdominal surgery	2006–2007 [30, 45]	This block is a novel analgesic technique designed to block abdominal wall neural afferents via bilateral injections in the lumbar triangles of Petit. It is relatively simple and used in patients undergoing abdominal surgery [32]. It was reintroduced and gained significant popularity in its current versions with ubiquitous use of ultrasound guidance.
4	Ultrasound guidance for peripheral nerve blocks	1991–1994 [43, 44]	Ultrasound guidance for peripheral nerve blocks was a natural continuation of the development of interventional and neural ultrasonography [38–40]. The use of Doppler ultrasound to locate the subclavian artery for a brachial plexus block [41], and ultrasound-guided neurolytic blocks [42] are closely associated with the initial use of ultrasound-guided anesthetic perineural injections for peripheral nerve blocks.
5	Femoral nerve block to provide analgesia in knee surgery	1984–1987 [53, 54]	Was developed as an extension of femoral nerve block used in the knee injuries [55].
6	Effect of perioperative epidural analgesia combined with general anesthesia on mortality in cardiac surgery	1989–1990 [56, 57]	The use of perioperative epidural anesthesia and analgesia versus general anesthesia with systemic opioids in mostly orthopedic surgery reduced overall mortality by approximately 30% [58]. This is the basis for expectations that perioperative thoracic epidural analgesia combined with general anesthesia can decrease mortality in cardiac and thoracic surgery.
7	Preemptive effect of epidural analgesics	1994 [59, 60]	The concept of preemptive analgesia was formulated by Crile at the beginning of the previous century on the basis of clinical observations [61]. The revival of this idea was associated with a series of animal studies started by Woolf (1983) [62, 63].
8	Spinal anesthesia is associated with lower intraoperative blood loss	1975–1980 [64, 65]	Induced hypotension provided with vasodilator agents [66] was previously often used for reduction of blood loss during surgery. Hypotension induced by spinal anesthesia was one of the forms of hypotensive anesthesia, it became more common technique to reduce the intraoperative blood loss.

## Discussion

Our assessment of research advances in regional/local anesthesia through the prism of meta-analysis revealed seven recent developments that passed the proof-of-concept step (Table 6). However, the positive conclusion regarding the most important factor – real clinical benefits – was reached only with one development – “ultrasound guidance for upper and lower limb nerve blocks”. Validation for real clinical benefits with other developments remains uncertain.

As far as the proof-of-concept step is concerned, statistical validation revealed robust results with seven of the eight analyzed outcomes: *p*-values were well beyond the fragile 0.05 level [5]. Although with one of the outcomes – “the effect of perioperative epidural analgesia combined with general anesthesia on mortality in cardiac surgery” – the *p*-value was insufficient (*p* = 0.72). These results were assessed as negative; however, they were not completely excluded from our presentation because of the extremely high number of the participating patients with results – 2877, and the common perception among anesthesiologists that overall mortality is lower in patients receiving neuraxial blockade [31].

Among the three summarized criteria for statistical assessment (*p*-value, effect size, and heterogeneity) heterogeneity (across various RCTs results) was usually the least reliable factor in the statistical assessment of outcomes. The exception was only with “ultrasound guidance for upper and lower limb nerve blocks,” where heterogeneity was low (Table 4). The high heterogeneity observed with all other analyzed outcomes suggests that there were many differences (outcome variables, different surgical procedures, etc.) between summarized RCTs. Such differences are bound to provide multiple noise factors [32]. This is troublesome because it means that the effect of the treatment in any particular setting is unpredictable.

Positive conclusion on real clinical benefits was achieved only with one outcome – “ultrasound guidance for upper and lower limb nerve blocks”. Our findings support (what is now a well established notion) that statistically significant changes, especially in pain, do not necessarily equate clinical importance [9, 10, 33]. Challenges in determining the clinical significance of any change or difference in an outcome measure have led to the development a new type of indicator – minimal clinically important difference (MCID). The MCID is the smallest change or difference in an outcome measure that is perceived as beneficial and would lead to a change in the patient’s medical management [11, 34]. For acute pain the determination of such an index is rather complicated. Bird and Dickson [10] explored the concept of MCID in pain using a visual analog scale. They concluded that in patients with moderate pain intensity (VAS score of 34–66 mm) the minimal clinically significant change in pain is 19 mm (patient’s perception of ‘a little less pain’). In our two of the analyzed outcomes, one on thoracic paravertebral block and the other on femoral nerve block, the pain scores were used to assess its intensity. In both cases, the changes in the scores had *p*-values of high significance (*p* < 0.0001); at the same time the degrees of these changes were much smaller than what is considered the minimal clinically important change: with thoracic paravertebral block it was *0.9 (NRS 0–10*; CI – 1*.3*; 5.0) and with femoral block it was 7.2 mm (VAS 0–100; CI – 9.3; 5.1), both much lower than the *value* determined by Bird and Dickson [10]. It should be mentioned that with these two outcomes there was not only a decrease in pain scores, but also a decrease in opioid consumption, another index of analgesia. Simultaneous changes in pain intensity and opioid consumption represent a difficulty in the interpretation of quantitative analgesic changes: one outcome measure interferes with exact assessment of the degree of changes in the other outcome measure [35]. Thus, relatively small changes in pain score may underrepresent changes in analgesia if paralleled by a decrease in opioid consumption.

“Ultrasound guidance for upper and lower limb nerve block” was the only advancement with positive assessment of real clinical benefits. However, even this advancement did not meet the criterion of positive “real-world evidence,” i.e., that this advancement will be useful in a large, more-inclusive population of patients, providers, and health care delivery systems or settings that reflect actual use in practice [36, 37]. For example, the authors of the assessed meta-analysis [17] were unable to determine whether their positive finding reflected the use of ultrasound only in experienced hands which represents only a fraction of real-world practice.

Our assessment of analyzed advancements in regional/local anesthesia over the past 30 years indicates that in almost all areas progress was incremental. The most valuable achievement in terms of clinical importance was with ultrasound guidance of peripheral nerve blocks. The incremental nature of the development in this area is clear when compared with earlier developments (Table 7). Ultrasound guidance for peripheral nerve blocks was a natural continuation of the development of interventional and neural ultrasonography [38–40]. The use of Doppler ulstrasound to locate the subclavian artery for a brachial plexus block [41], and ultrasound-guided neurolytic blocks [42] are closely associated with the initial use of ultrasound-guided anesthetic perineural injections for peripheral nerve blocks, which was first reported in 1991–1994 [43, 44]. Within this analysis, the most original regional anesthesia technique advancement is the introduction of the transversus abdominis plane block [30, 45], yet the clinical importance of this block awaits careful assessment [32].

It is of interest to compare the conclusions on research progress in the area of regional/local anesthesia assessed via meta-analyses with those based on the assessments using scientometric indices. Our previous evaluation of different anesthetics and techniques of their administration using a number of scientometric indices indicated that, for the past 30 years, there were no new advances, except for ultrasound guidance, that have produced changes in these indices indicating real progress [1]. In addition, there were more academic publications related to regional/local anesthetics than to general anesthetics. Similar to the scientometric analysis, our assessment of research advances in regional/local anesthesia through the prism of meta-analysis indicated that advances were few and only incremental in almost all cases. The most significant of these developments was the introductoion of ultrasound guidance for peripheral nerve blocks. It is worth mentioning that the scientometric analysis of this technique demonstrated that the index of change (IC) for ultrasound-guided block was extremely high for all three studied 5-year periods – 1999-2003, 2004–2008, and 2009–2013. At the same time, changes of the index of expectations (IE) were moderate [1]. Such a combination of changes in IC and IE is characteristic of a technique that is rapidly developing but whose degree of originality is rather limited.

## Conclusion

The assessment of recent advances in regional/local anesthesia, based on the evaluation of related meta-analyses analysis, revealed only incremental progress with mostly marginal benefits in several areas. The progress was the most notable with ultrasound guidance for some of peripheral nerve blocks.
